# Combining Multiple Resting-State fMRI Features during Classification: Optimized Frameworks and Their Application to Nicotine Addiction

**DOI:** 10.3389/fnhum.2017.00362

**Published:** 2017-07-12

**Authors:** Xiaoyu Ding, Yihong Yang, Elliot A. Stein, Thomas J. Ross

**Affiliations:** Neuroimaging Research Branch, Intramural Research Program, National Institute on Drug Abuse, National Institutes of Health Baltimore, MD, United States

**Keywords:** feature combination, kernel combination, classifier combination, resting-state fMRI, nicotine addiction, support vector machine

## Abstract

Machine learning techniques have been applied to resting-state fMRI data to predict neurological or neuropsychiatric disease states. Existing studies have used either a single type of resting-state feature or a few feature types (<4) in the prediction model. However, resting-state data can be processed in many different ways, yielding different feature types containing complementary and/or novel information, leaving uncertain the most informative features to provide to the classifier. In this study, multiple resting-state features were calculated from two main analytical categories: local measures and network measures. Feature selection was adopted using an optimized grid-search approach selecting top ranked features from statistical tests. We then tested three optimized frameworks: feature combination, kernel combination, and classifier combination, all using the support vector machine as an elementary classifier, to combine these resting-state feature types. When applied to nicotine addiction, with a cohort size of 100 smokers and 100 non-smokers, via a 10-fold cross-validation procedure, the feature combination and the classifier combination achieved an accuracy of 75.5%, while the kernel combination achieved a 73.0% accuracy; all three combination frameworks improved classification performance compared to the single feature type based results (best accuracy 70.5%). This study not only reveals the discriminative power of resting-state data, but also demonstrates the efficiency of combining multiple features from one data phenotype to improve classification performance.

## Introduction

Machine learning techniques are playing an increasingly important role in neuroscience research to explore various brain functions (Klöppel et al., 2012; Richiardi et al., 2013; Sundermann et al., 2014; Gabrieli et al., 2015). They have been applied to neuroimaging data to predict group membership, which may lead to brain-based biomarkers of disease (Chen and Herskovits, 2010; Wang et al., 2010; Zhang and Shen, 2012; Hart et al., 2014; Pariyadath et al., 2014; Ding et al., 2015; Jie et al., 2015; Libero et al., 2015; Liu et al., 2015; Moradi et al., 2015; Suk et al., 2015; Arbabshirani et al., 2016). A prominent advantage of machine learning algorithms is that they learn a computational model from exemplar inputs, which can later be applied to new unknown samples to make predictions or decisions. Moreover, discriminative features selected by machine learning techniques can uncover multivariate relationships beyond those found by univariate analysis such as simple statistical tests. For neuroimaging data, the model is usually evaluated using a cross-validation (CV) procedure, in which a set of data is split into complementary subsets separately used for training and testing the model (Hirsch, 1991; Wolfers et al., 2015).

Support vector machine (SVM) is one of the most popular machine learning algorithms that has been applied to neuroimaging data (for a review, see Orrù et al., 2012). In binary classification, given a set of training samples, each with a label marked for its category, a SVM constructs a separating hyperplane that maximizes the margin between samples (Cortes and Vapnik, 1995; Burges, 1998). However, frequently the sets are not linearly separable in the original input space. In this case, these samples are first mapped into a higher dimensional space using a kernel function, which presumably makes the separation easier in the transformed space. A commonly adopted kernel function is the Gaussian radial basis function (RBF) that maps the input samples into a Hilbert space, corresponding to a non-linear SVM called RBF kernel SVM (RBF-SVM) (Burges, 1998).

Resting-state fMRI is a functional brain imaging method that measures spontaneous fluctuations in blood-oxygen-level dependent (BOLD) signals that occur in the absence of an explicit task (for a review, see Lee et al., 2013). Thus, the resting-state approach is ideal to examine brain function in patients who may experience difficulty in performing tasks. Resting-state data are widely investigated using machine learning approaches (Deshpande et al., 2010; Shen et al., 2010; Dai et al., 2012; Eloyan et al., 2012; Zeng et al., 2014; Iidaka, 2015; Liu et al., 2015; Rehme et al., 2015). For example, our group previously applied SVM-based classification to resting-state functional connectivity (rsFC) data from 21 smokers and 21 non-smokers to successfully predict smoking status (Pariyadath et al., 2014). Three network characteristics, including network representativeness, within network connectivity, and between network connectivity were tested, separately. Among these, within network connectivity offered maximal information for predicting smoking status with an accuracy of 78.6% using leave-one-out cross-validation (LOOCV).

As in the above example, most studies that applied machine learning techniques to resting-state data used either a single type of resting-state feature (Shen et al., 2010; Zeng et al., 2014; Iidaka, 2015; Liu et al., 2015; Rehme et al., 2015) or just a few feature types (<4) (Deshpande et al., 2010; Dai et al., 2012) to do prediction. However, resting-state data can be processed in many different ways, yielding different feature types containing complementary and/or novel information. When applied to brain disorders using machine learning, these feature types may also provide disparate discriminative information. Furthermore, they may be combined in different ways, potentially leading to an improved model performance.

The purpose of the present methodological study was to determine the optimal resting-state feature types to enter into several classification models. Multiple resting-state feature types were calculated from two main data categories: local measures and network measures. Feature selection was adopted using an optimized grid-search approach selecting top ranked features from two-sample *t*-tests. We then implemented three optimized classification frameworks: feature combination, kernel combination, and classifier combination, all using the RBF-SVM as an elementary classifier.

## Materials and methods

### Participants

In order to evaluate their performance, the three frameworks were applied to existing nicotine addiction data from our lab. One hundred cigarette smokers and 100 non-smoking healthy control participants matched on age and gender (see Table 1 for demographics) were enrolled under several protocols approved by the Institutional Review Board of the National Institute on Drug Abuse Intramural Research Program (NIDA-IRP). Smokers were not currently trying to quit or seeking smoking cessation treatment and were allowed to smoke *ad libitum* prior to the scan session. Controls were included if they had smoked fewer than 25 cigarettes in their lifetime and none in the past year. Potential participants were assessed with a comprehensive medical history and physical exam, general urine and blood laboratory panels, a computerized Structured Clinical Interview for DSM-IV with follow-up clinical interview, and a drug use survey. Participants were excluded if they had any major medical illness, history of neurological or psychiatric disorders, or current or past dependence on any drug other than nicotine. All participants provided written informed consent approved by the NIDA-IRP IRB and received monetary compensation for their participation.

**Table 1:** Demographics of the participants.

	**Smokers**	**Non-smokers**
Number	100	100
Age	31.9 ± 9.5	32.6 ± 9.9
Gender	53 M, 47 F	53 M, 47 F
FTND	5.3 ± 1.9	–
CPD	18.2 ± 6.8	–
Smoking years	15.3 ± 9.4	–
Lifetime usage	14.5 ± 11.9	–

*P > 0.6 on age between smokers and non-smokers*.

*FTND: Fagerström Test for Nicotine Dependence*.

*CPD: cigarettes per day*.

*Lifetime usage: measured in pack-years (= CPD × smoking years/20)*.

*M/F: male/female*.

*Age, FTND, CPD, smoking years and lifetime usage are calculated in mean ± SD*.

### Data acquisition and preprocessing

Functional MRI data were collected at the NIDA-IRP on a 3T Siemens Allegra MRI scanner (Erlangen, Germany) equipped with a standard radio frequency birdcage head coil. During the resting-state scanning, 39 slices, without interslice gap, 30° from AC-PC, were prescribed to cover the whole brain. The resting-state data were acquired using a single-shot gradient echo-planar imaging (EPI) sequence with repetition time (TR) of 2,000 ms, echo time (TE) of 27 ms, flip angle (FA) = 80°, field of view (FOV) of 220 × 220 mm, and acquisition matrix of 64 × 64, resulting in 300 volumes for each subject. For registration purposes, high-resolution anatomical images were acquired using a 3D magnetization prepared rapid gradient-echo (MPRAGE) T1-weighted sequence in 1 mm^3^ isotropic voxels (TR = 2,500 ms, TE = 4.38 ms, FA = 8°).

Data preprocessing were conducted in AFNI (Cox, 1996) including slice timing and head motion correction. Data were then spatially normalized to a template in Talairach space to a resampled resolution of 3 × 3 × 3 mm^3^. White matter (WM) and cerebrospinal fluid (CSF) signals, originating presumably from such systemic effects as respiration and cardiac-induced pulsations, were accounted for individually by extracting the first three principal components from a WM time course ensemble and the first three principal components from a CSF time course ensemble (Behzadi et al., 2007). Here the WM and CSF masks were generated by segmenting the high resolution structural images in AFNI (3dSeg) and down sampling the obtained WM and CSF masks to the same resolution as the functional data. In addition to these physiological regressors, time courses of the six motion parameters also served as uninteresting covariates. The data were temporally band-pass filtered (0.01–0.1 Hz) and uninteresting covariates were removed simultaneously using 3dBandpass in AFNI. Next the data were spatially smoothed with an 8 mm full-width half-maximum (FWHM) Gaussian kernel to increase spatial signal to noise ratio. Finally, data were censored for motion with a threshold of 0.35 for a frame-to-frame change in Euclidean norm of the six motion parameters (Power et al., 2012, 2014, 2015). Two smokers whose censored volumes exceeded 1/3 of the original time series were removed from further analysis (The excluded subjects are not included in Table 1). All remaining subjects had at least 80% of their data retained. Further, there was no significant difference (*p* = 0.995) between groups on the numbers of time points that were censored. Most of the feature sets were calculated using the censored data, however, as noted in the following section, some of them utilized the uncensored data.

### Feature extraction

Since resting-state data can be processed in many different ways yielding different feature types, we calculated multiple resting-state feature types from two main analysis categories: local measures and network measures. These feature types are detailed below. Our motivation for extracting these feature types were 2-fold: first, these are among the most common ways that resting-state data are analyzed; second, it has been demonstrated that nicotine dependent individuals show abnormalities in many of these resting-state features, with the expectation therefore of maximizing our ability to separate the groups (Sutherland et al., 2012; Ding and Lee, 2013; Fedota and Stein, 2015; Wu et al., 2015).

### Local measures

Four local measures including the amplitude of low frequency fluctuations, regional homogeneity, voxel-mirrored homotopic connectivity, and functional connectivity strength, were calculated. We consider these as local measures given that they represent values in each brain region (i.e., nodes in graph theory).

#### Amplitude of low frequency fluctuations (ALFF)

ALFF measures regional spontaneous fluctuations in BOLD signal intensity in the resting-state brain. Briefly, the time series of preprocessed but uncensored data was transformed to the frequency domain using a fast Fourier transform (FFT). The square root of the power spectrum was calculated at each frequency and then averaged across 0.01–0.1 Hz at each voxel. This averaged square root was taken as the ALFF (Zang et al., 2007). For standardization purpose (i.e., reducing the global effects of variability across subjects), the ALFF of each voxel was divided by the global mean ALFF value for each subject.

#### Regional homogeneity (ReHo)

Based on the hypothesis that intrinsic brain activity is manifest by clusters of voxels rather than single voxels, ReHo evaluates the degree of regional similarity or synchronization of fMRI time courses (Zang et al., 2004). It is defined as the Kendall's coefficient concordance (KCC) (Kendall and Gibbons, 1990) of time series within a given voxel and its nearest neighbors. In the current analyses, the number of neighboring voxels was set to 26, which included voxels on the faces, edges, and the corners of a given voxel. For standardization purpose, as used in the ALFF calculation, the ReHo of each voxel was divided by the global mean ReHo value for each subject.

#### Voxel-mirrored homotopic connectivity (VMHC)

Functional homotopy, the synchrony in spontaneous activity between geometrically corresponding interhemispheric regions, is a fundamental characteristic of the brain's functional architecture (Salvador et al., 2005). It can be quantified by calculating the Pearson correlation coefficient between each voxel's time series and that of its symmetric inter-hemispheric counterpart (Zuo et al., 2010). Correlation values were then transformed by Fisher's Z-transformation $\left(z=\frac{1}{2}log\left(\frac{1+r}{1-r}\right)\right)$ to approach a normal distribution.

#### Functional connectivity strength (FCS)

FCS at a voxel is defined as the average functional connectivity (FC) between that given voxel and all other voxels in the brain, i.e., $FCS_{i}=\frac{1}{N-1}\sum_{j\neq i}FC_{ij}$ (Liang et al., 2013). In this experiment, we only considered FCS within gray matter (GM) voxels. Pearson correlation coefficients between each voxel and all other voxels in an individual's GM mask were calculated and transformed into z-scores using Fisher's Z-transformation; FCS maps were then computed. Here the GM mask was derived from the segmentation step during data preprocessing.

We used the Resting-State fMRI Data Analysis Toolkit (Song et al., 2011) to calculate the ALFF, ReHo, and VMHC maps. For all local measures, mean values were extracted from individuals using the 116 region standard Automated Anatomical Labeling (AAL) template (Tzourio-Mazoyer et al., 2002) which served as input features for the classifiers. The use of the AAL template aimed to reduce feature dimensions and improve signal-to-noise ratio.

### Network measures

We categorize the following as network measures given that they characterize the relationship between pairs of brain regions (i.e., edges in graph theory). Two kinds of network measures were considered in this study: One was seed-based brain networks, measuring the correlation between one voxel cluster (i.e., the seed) and all other voxels in the brain; the other, including temporal correlation and Granger causality, was the interaction of signals between all pairs of AAL regions.

#### Seed-based brain networks

Seed-based methods were applied using AFNI to extract five widely studied large-scale brain networks: the default-mode network (DMN), executive-control network (ECN), salience network (SN), striatum network (StrN), and limbic network (LN). Notably, the DMN, ECN, and SN have been implicated to work in an interacting fashion, including in nicotine dependence (Sridharan et al., 2008; Bressler and Menon, 2010; Bonnelle et al., 2012; Sutherland et al., 2012; Jilka et al., 2014; Lerman et al., 2014; Liang et al., 2015; Uddin, 2015). Additionally, the StrN and LN are two networks putatively related to drug addiction (Kelley and Berridge, 2002; David et al., 2005; Everitt and Robbins, 2005; Gu et al., 2010; Janes et al., 2012). Seed regions were defined by placing bilateral 3 mm radius spherical regions of interest (ROIs) in the posterior cingulate cortex (PCC) as an exemplar constituent of the DMN (Greicius et al., 2003), the dorsal lateral prefrontal cortex (dlPFC) for the ECN (Seeley et al., 2007), the insula for the SN (Seeley et al., 2007), the caudate for the StrN (Di Martino et al., 2008), and the amygdala for the LN (Gu et al., 2010); see Table 2 for center coordinates of seeds. For each brain network seed, a reference time course was generated by averaging the time course from all voxels within the ROI. Subsequently, a correlation coefficient (CC) map was obtained by correlating each voxel's time course with the corresponding reference time course. The CC maps were then transformed by Fisher's Z-transformation into z-score maps. Finally, these z-scored brain network maps were partitioned into 116 ROIs using the standard AAL atlas, and mean values within each AAL region served as input features for the classifiers.

**Table 2:** Seeds locations used to define brain networks.

**Brain networks**	**Seed location**	**Center (Talairach coordinates)**
Default-mode network (DMN)	PCC	(±2, −51, 27)
Executive-control network (ECN)	dlPFC	(±43, 36, 21)
Salience network (SN)	Insula	(±40, 6, −6)
Limbic network (LN)	Amygdala	(±23, -5, −15)
Striatum network (StrN)	Caudate	(±8, 6, −4)

#### Temporal correlation (TC)

Functional connectivity refers to the functionally integrated relationship between different brain regions regardless of the apparent physical connectedness (Friston, 2011). One definition is the TC between spatially remote neurophysiological events (Biswal et al., 1995). In contrast to the above seed-based network measures, which compute the functional connectivity between a well-defined, *a priori* hypothesized ROI and all other voxels, the TC here is calculated pairwise using mean time series extracted from standard AAL template regions. Pearson correlation coefficients were computed between mean time series extracted from the 116 standard AAL regions, and transformed into z-scores using Fisher's Z-transformation. Due to symmetry, we only took the lower triangle z-score matrices as our input features.

#### Granger causality (GC)

In contrast to temporal correlation, one may attempt to measure causal influence exerted by one neuronal system onto another (Goebel et al., 2003; Friston, 2011). Granger causality analysis (GCA) has been proposed to estimate the causal interactions of information flow. It models one directional causality among multiple time series based on a vector autoregression (VAR) model (Seth, 2005). When the model's residual error reaches the minimum, an *F*-test is used to estimate the statistical significance of the estimated model. A higher *F*-score means a stronger prediction of GC between two time series. We employed a Matlab toolbox for GCA (Seth, 2010) to calculate the GC between mean time series of uncensored data within 116 regions standard AAL atlas. The VAR model order was estimated using Akaike information criterion (AIC) (Burnham and Anderson, 2004). Resulting *F*-score matrices were treated as input features. It should be noted that the use of GCA applied to neuroimaging data is controversial (Friston et al., 2013). However, we make no claims of its ability to determine any causal relationship between regions; it is merely another feature that may convey complementary information to improve group discrimination classification accuracy (Deshpande et al., 2010).

In this study, we used the AAL template, which is an anatomical atlas based template, to define our ROIs from which features were extracted from each resting-state feature type. Another common way to process resting state data is to use independent components analysis (ICA) to define brain networks. Some studies extracted ICA components from the respective groups to conduct machine learning (Van Waarde et al., 2015) or resting state functional connectivity (Cerliani et al., 2015) analysis. In an effort to compare the AAL template with ICA-derived regions, we additionally applied a network-based ROI template approach where networks were generated from publicly available group ICA maps (Smith et al., 2009). Details are described in Supplementary Materials.

### Feature selection using grid-search

In contrast to studies that selected discriminative features lower than a statistical threshold (Fan et al., 2007; Deshpande et al., 2010; Dai et al., 2012; Feis et al., 2013; Hart et al., 2014), we determined the optimal percentage of reserved features using a grid-search method for the three frameworks described below. Specifically, for each feature type, with the aid of an inner 10-fold CV, features were first sorted based on their *T*-scores using a two-sample *t*-test (Pereira et al., 2009; Chu et al., 2012; Mwangi et al., 2014). An RBF-SVM was then used to search for an optimized feature size within percentage values(1, 5, 10, 15, 20, 25, 30, 35, 40, 45, and 50%) of the sorted features. Thus, in the inner fold, the optimized feature size varied for different feature types and for different folds, which means that the function of performance along with the size of reserved features changed in each inner fold for each feature type. The LIBSVM toolbox (Chang and Lin, 2011) was used for all classification procedures. Two hyperparameters including the regularization constant *C* and Gaussian kernel parameter γ in the RBF-SVM were optimized using a nested 10-fold CV among the values of 2^*N*^ (*N* from −4 to 6 for *C* and from −10 to 3 for γ). In addition to the optimized feature size, we also recorded its corresponding accuracy in the inner 10-fold CV for later use.

### Three optimized frameworks

To address the combinatorial options of the multiple feature types, we implemented three optimized frameworks: feature combination, kernel combination, and classifier combination.

#### Feature combination framework

**Figure 1:**
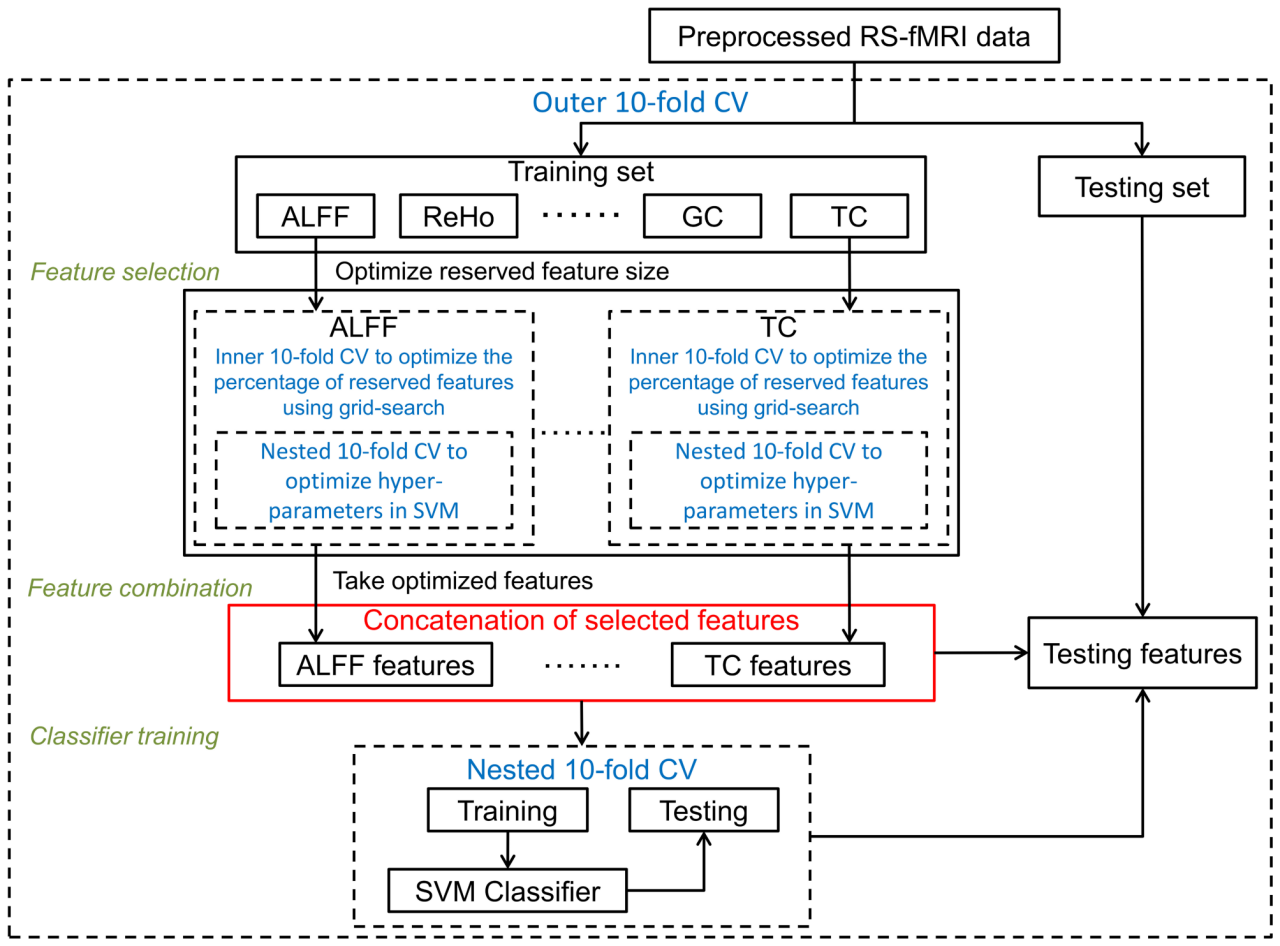
Optimized framework of feature combination: after feature selection, optimized features were concatenated to serve the classifier training. Cross-validation parts are in blue font and dashed lines. To distinguish from the other two frameworks, the feature concatenation part is illustrated in red.

The feature combination framework illustrated in Figure 1 performed a multi-feature type combination before classifier training. In this framework, selected features from each type were concatenated into a row vector and input to an RBF-SVM classifier. Hyperparameters *C* and γ in the RBF-SVM were optimized using a nested 10-fold CV among the values of 2^*N*^ (*N* from −4 to 6 for *C* and from −10 to 3 for γ).

#### Kernel combination framework

As mentioned in the Introduction, features are more likely to be linearly separable when they are projected into a higher dimensional space through a kernel-induced implicit mapping function. A well-known property of kernels is that they can be combined via linear operations to yield a new valid kernel. Let ${\text{x}}_{i}^{\left(m\right)}$ denote a feature vector in the *m*-th feature type of the *i*-th sample whose class label is *y*_*i*_ ∈ {−1, 1}. Multi-kernel SVM aims to solve the following primal problem:

(1) $$\begin{array}{l} \underset{w^{\left(m\right)},b,\varepsilon_{i}}{\min}\frac{1}{2}\sum_{m=1}^{M}\beta_{m}||w^{\left(m\right)}|{|}^{2}+C\sum_{i=1}^{n}\varepsilon_{i}\\ s.t.\;y_{i}\left(\sum_{\left(m=1\right)}^{M}\beta_{m}\left({\left(w^{\left(m\right)}\right)}^{T}K^{\left(m\right)}\left(x_{i}^{\left(m\right)}\right)+b\right)\right)\geq 1-\\ \varepsilon_{i},\varepsilon_{i}\geq 0,\beta_{m}\geq 0,i=1\ldots n \end{array}$$

Here, ***w***^(*m*)^, ***K***^(*m*)^, β_*m*_, ε_*i*_, *C*, and *b* denote, respectively, the weight vector of hyperplane, the kernel-induced mapping function, the combining weight on the kernel, the non-negative slack variable, the trade-off, and the offset of the hyperplane. A more detailed description of multi-kernel SVM can be found in (Gonen and Alpaydin, 2011). In this experiment, selected features from each type were projected into a higher dimensional space using RBF as the kernel mapping function.

**Figure 2:**
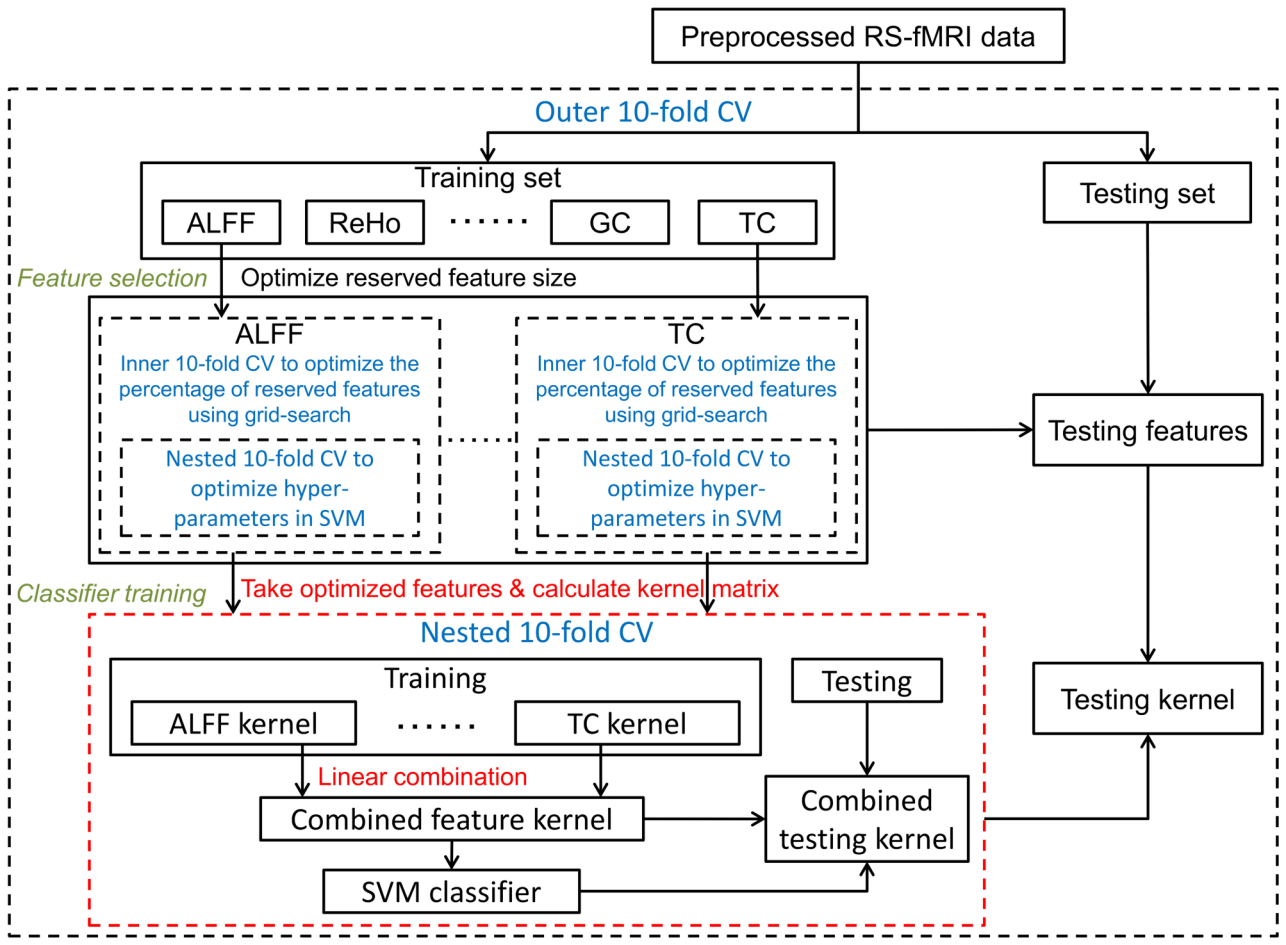
Optimized framework of kernel combination: kernel matrices were calculated separately on each optimized feature set, and were then linearly combined as a final kernel. Cross-validation parts are in blue font and dashed lines. To distinguish it from the other two frameworks, the kernel combination part is illustrated in red.

Since the main idea of multi-kernel SVM is to first construct an individual kernel for each feature type and then train a mixed kernel based on the linear combination of all individual kernels (Zhang D. et al., 2011), similar to Gonen and Alpaydin (2011); Zhang D. et al. (2011) and Zhang and Shen (2012), we added a constraint $\overset{M}{\underset{m=1}{\sum }}\beta_{m}=1$ to the kernel combining weights. Considering the large number of feature types (11 in total) in our experiment, and in contrast to prior work utilizing this technique (Zhang D. et al., 2011; Zhang and Shen, 2012) that applied a coarse grid-search method to determine the combining weights for only three modalities, we chose a heuristic approach in which the recorded accuracy of each feature type from the above feature selection procedure was used to choose the combining weights in the following form:

(2) $$\{\begin{array}{c} \beta_{m}=\frac{Acc_{m}-0.5}{\sum_{i=1}^{M}\left(Acc_{i}-0.5\right)};\;if\;Acc_{m}>0.5,\;and\;for\;all\;Acc_{i}>0.5\\ \beta_{m}=0;\;if\;Acc_{m}\leq 0.5 \end{array}$$

where *Acc*_*m*_ denotes the accuracy of *m*-th feature type via inner 10-fold CV in the feature selection. The other hyperparameters in the multi-kernel SVM were optimized using the above described grid-search method. The kernel combination framework is illustrated in Figure 2.

#### Classifier combination framework

**Figure 3:**
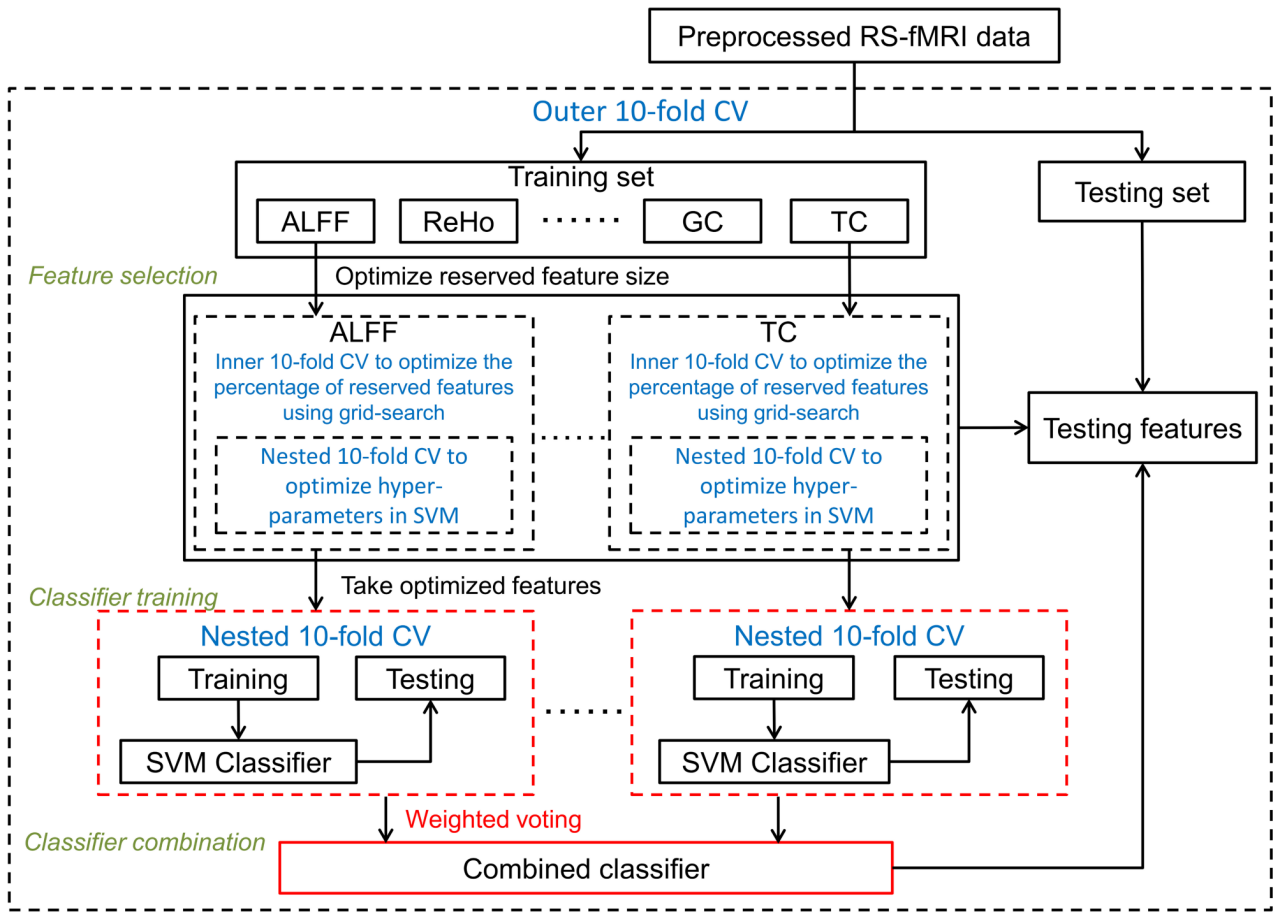
Optimized framework of classifier combination: classifiers were trained separately on each optimized feature set, and a final classifier was then combined using weighted voting. Cross-validation parts are in blue font and dashed lines. To distinguish this combination from other the two frameworks, the classifier combination part is shown in red.

Discriminative information from multiple feature types can also be combined after classifier training, which is the basis of our classifier combination framework (see Figure 3). In this framework, selected features from each type were input to a RBF-SVM classifier. Hyperparameters were optimized as described above. Let *f*_*m*_(*x*_*i*_) be an output decision value of the SVM classifier on *m*-th feature type for *i*-th sample, a final classifier was then combined using a weighted voting approach:

(3) $$y_{i}=sgn\left(\sum_{\left(m=1\right)}^{M}\beta_{m}f_{m}\left(x_{i}\right)\right),s.t.,\sum_{m=1}^{M}\beta_{m}=1$$

Here, β_*m*_ is the classifier combining weight for *m*-th feature type, and was determined using the same weighting scheme described above.

#### Cross-validation

As illustrated in Figures 1–3, all three frameworks were evaluated using a balanced outer 10-fold CV procedure. That is, in each outer trial, 10 smokers and 10 non-smokers were excluded before feature selection (i.e., they were left out of the whole analysis) for testing the classifier that was trained using all other subjects. The classification quality was assessed by the following five quantities:

(4) Sensitivity=TP/(TP+FN)

(5) Specificity=TN/(TN+FP)

(6) Accuracy=(TP+TN)/(TP+FN+TN+FP)

(7) Precision=TP/(TP+FP)

(8) F score=2TP/(2TP+FP+FN)

Here, TP, FN, TN, and FP denote, respectively, the number of smokers correctly classified, the number of smokers predicted to be non-smokers, the number of non-smokers correctly classified, and the number of non-smokers predicted to be smokers. Specifically, sensitivity, also called the true positive rate, measures the proportion of smokers that are correctly identified as such; while specificity, also called the true negative rate, measures the proportion of non-smokers that are correctly identified as such. Precision, also called positive predictive value, is the proportion of smokers that are identified as such; and F score is the harmonic mean of precision and sensitivity. We did not employ the receiver operating characteristic (ROC) calculation to assess our frameworks; since in our classifier combination framework, a SVM classifier was trained on each feature set, it would be unreasonable to move the cut-off thresholds in the same range for each SVM to plot a ROC curve.

Finally, we performed significance analysis on the selected feature maps: For each resting-state feature type in feature selection, we recorded the percentage of features that was reserved in each fold from the three frameworks (30-folds in total). To determine the threshold of significant ROIs for each feature type, we first randomly chose ROIs according to the recorded percentage (i.e., if 5% of the regions were retained for that fold, we randomly choose 5% of the ROIs to assess significance). We then calculated the number of times that an ROI was randomly selected among all 30-folds. This whole process was repeated 1,000 times to derive an empirical null distribution. The actual data were thresholded at *P* < 0.05 based upon the empirical null.

## Results

### Classification performance

Classification results of the three tested frameworks are shown in Table 3. Using nicotine dependence as a model system and using all feature types, the three approaches overall yielded very similar results; the classifier combination and the feature combination frameworks reached an accuracy of 75.5%, while the kernel combination achieved a 73.0% accuracy. As a comparison, the discriminative ability of each feature type was tested individually (see Table 4), where the RBF-SVM classifiers were performed separately on each type using the same feature selection and hyperparameter optimizing framework. As a single feature type, TC achieved the highest accuracy of 70.5%, but most of the other feature types individually only performed at slightly above chance with the exception of GC. Notably, all proposed combination frameworks improved the classification accuracy over any single feature type. Since GC showed the lowest performance (accuracy = 49.0%) among the single feature types, we implemented the three combination frameworks following GC elimination. Somewhat paradoxically, the accuracy of all three frameworks slightly decreased (see Table 3) when excluding this feature type that performed worse than chance on its own, indicating that even the worst feature contributed some information to the combination frameworks.

**Table 3:** Classification results of the three tested frameworks.

		**Sensitivity (%)**	**Specificity (%)**	**Accuracy (%)**	**Precision (%)**	***F* score (%)**
Feature combination	All features	76.0	75.0	75.5	75.2	75.6
	Without GC	73.0	71.0	72.0	71.6	72.3
Kernel combination	All features	77.0	69.0	73.0	71.3	74.0
	Without GC	74.0	68.0	71.0	69.8	71.8
Classifier combination	All features	79.0	72.0	75.5	73.8	76.3
	Without GC	74.0	75.0	74.5	74.7	74.4

**Table 4:** Discriminative ability of individual feature types.

		**Sensitivity (%)**	**Specificity (%)**	**Accuracy (%)**	**Precision (%)**	***F* score (%)**
Local measures	ALFF	60.0	53.0	56.5	56.1	58.0
	ReHo	59.0	61.0	60.0	60.2	59.6
	FCS	46.0	67.0	56.5	58.2	51.4
	VMHC	46.0	66.0	56.0	57.5	51.1
Network measures	DMN	62.0	64.0	63.0	63.3	62.6
	ECN	58.0	42.0	50.0	50.0	53.7
	LN	66.0	41.0	53.5	52.8	58.7
	SN	54.0	57.0	55.5	55.7	54.8
	StrN	61.0	54.0	57.5	57.0	58.9
	TC	76.0	65.0	70.5	68.5	72.0
	GC	48.0	50.0	49.0	49.0	48.5

Compared to the AAL template, all frameworks performed worse when using the ICA-based template (see Supplementary Materials for details). Given the superior classifier accuracy and brain coverage given by the AAL template, we report only those results in this manuscript.

**Figure 4:**
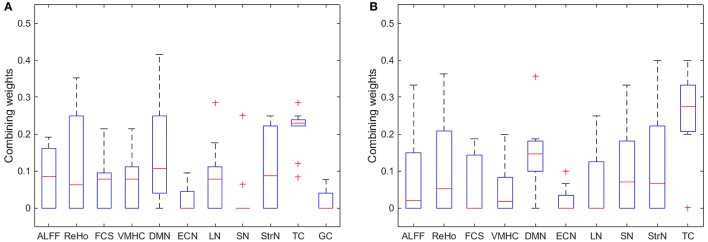
A box plot of β_m_ in Equation (2) for **(A)** all feature types, and **(B)** all remaining feature types after excluding GC.

A box plot of β_m_, used in the kernel combination and classifier combination frameworks, from Equation (2) shows that the feature type TC consistently had the highest weight, while other feature types performed similarly (see Figure 4). Also illustrated is that the GC was consistently the worst feature and was frequently weighted zero.

### Maps of significant ROIs

**Figure 5:**
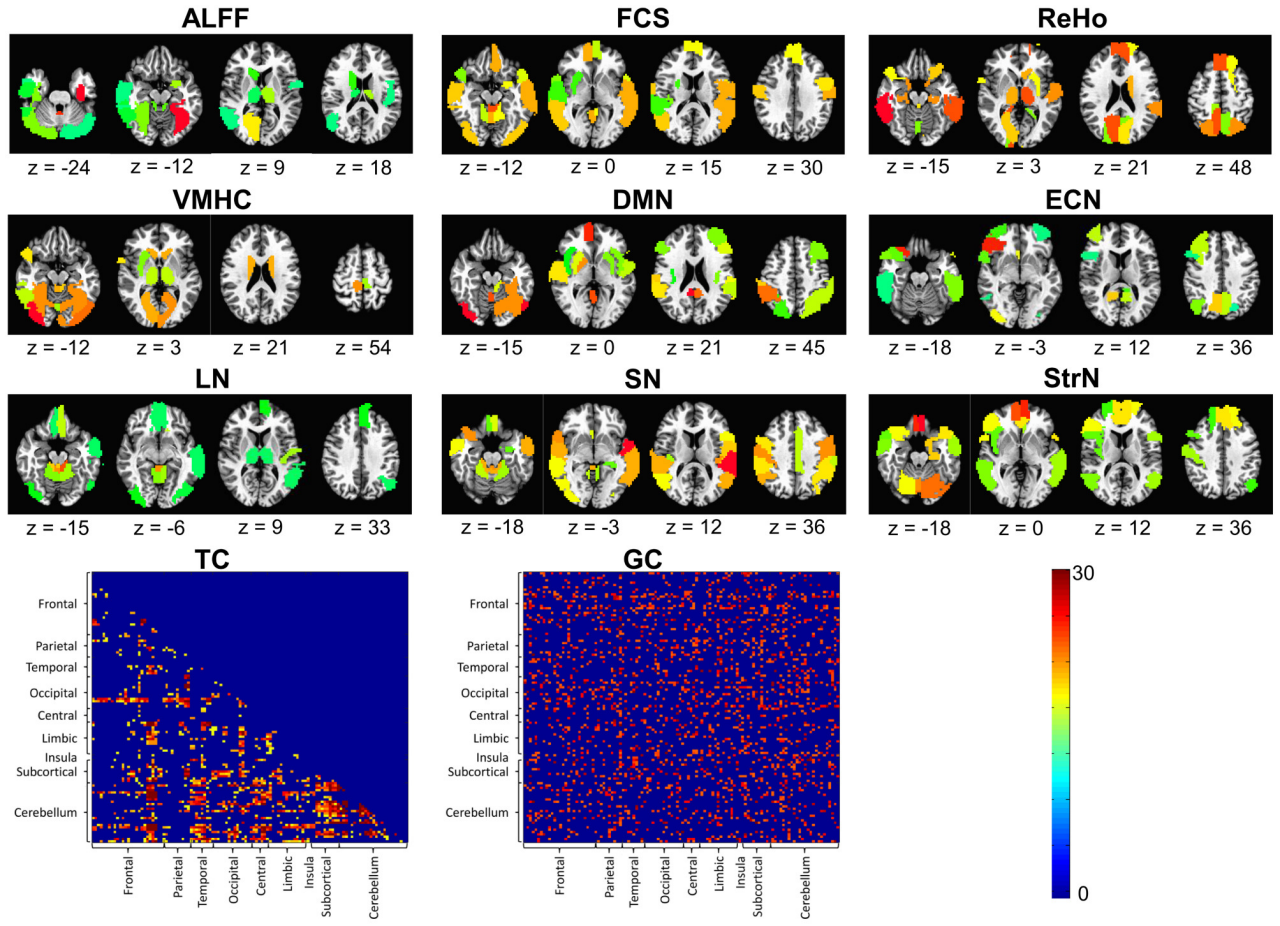
Significant feature maps for each of the applied resting state features: amplitude of low frequency fluctuations (ALFF), functional connectivity strength (FCS), regional homogeneity (ReHo), voxel-mirrored homotopic connectivity (VMHC), default-mode network (DMN), executive-control network (ECN), limbic network (LN), salience network (SN), striatum network (StrN), temporal correlation (TC), and Granger causality (GC). The color bar denotes the number of times that an ROI was selected in 10-fold cross-validation in the three combination frameworks (*p* < 0.05).

The threshold to reach significance for a given ROI (*P* < 0.05) was determined to be 9-folds for the ALFF, 15-folds for the FCS, 15-folds for the ReHo, 17-folds for the VMHC, 14-folds for the DMN, 10-folds for the ECN, 12-folds for the LN, 16-folds for the SN, 15-folds for the StrN, 17-folds for the TC, and 22-folds for the GC. Significant feature maps are shown in Figure 5. ROIs in the prefrontal cortex (PFC), subcortical regions (e.g., the thalamus, caudate, putamen), occipital lobe, and cerebellum were significant in the maps of many of the feature types. Among these, the thalamus was significant in both local (e.g., ALFF, ReHo, VMHC) and network measures (e.g., DMN, LN). Additionally, the TC between the subcortical regions and the frontal cortex as well as the cerebellum significantly differentiated smokers from non-smokers, suggesting abnormal functional connectivity between these regions in smokers.

## Discussion

In recent years, machine learning techniques have become widely applied to neuroimaging data to predict neurological and psychiatric disorders (Orrù et al., 2012; Wolfers et al., 2015; Arbabshirani et al., 2016), with the long-term goal to create complex brain-based biomarkers of disease status that could enormously benefit the treatment community. The scientific motivation of this study was to examine various techniques to combine multiple resting-state feature types so as to utilize their complementary information to maximize classification accuracy, thus this is first and foremost a methods paper. We proposed three frameworks addressing SVM classification using multi-type features: feature combination, kernel combination, and classifier combination. We chose nicotine addiction as an exemplar disease model system and tested the frameworks on 11 resting-state features consisting of both local and network measures to predict smoking status. All frameworks were validated using a 10-fold CV procedure, and all demonstrated an improvement over the classification performance using any one of the 11 single feature types.

We designed a grid search approach involving two-sample *t*-tests to sort the features for selection. Feature selection approaches are classified into “filter,” “wrapper,” and “embedded” methods (Pereira et al., 2009; Mwangi et al., 2014). The approach of the two-sample *t*-test that we chose falls under the “filter” category. Although some studies argue that *t*-test filtering is not stable and robust enough since it is only performed once using the training data (Venkataraman et al., 2010), it has been demonstrated that combining *t*-test filtering and atlas based ROI leads to significantly better accuracy than no feature selection when sample sizes are small (Chu et al., 2012). Another well-known available feature selection method is recursive feature elimination (RFE), which is a “wrapper” method. We chose, however, not to use it as it would be prohibitively computationally time consuming for our nested CV design with the number of features included herein because in RFE, features are sorted and the least discriminative feature is eliminated. This procedure is repeated iteratively until all features are tested.

Previously, (Pettersson-Yeo et al., 2014) also chose SVM as an elementary classifier on raw feature sets calculated from multiple modalities of imaging data to do multi-feature combination. In contrast to their method that combined raw feature maps to train classifiers without any feature selection, we designed an optimized feature selection procedure using a grid-search method on AAL atlas based ROI features ranked by two-sample *t*-tests. Advantages of using the AAL atlas are that it's anatomically based and the most commonly used ROI template; however, a potential limitation is that there is no direct physiological relationship between AAL regions and neurobiological processing units or nicotine addiction, making data interpretation neurobiologically difficult. In our hands, using smoking as a model neuropsychiatric disease (Leshner, 1997; Hasin et al., 2013), we observed improved classification using ROI based features over the raw voxel wise features (Ding et al., 2015), as the spatial averaging improves signal-to-noise. Using ROIs also benefited the classifier training in the combination frameworks, as it reduced feature dimension and improved the grid-search speed for eliminating less informative features.

Another major difference from Pettersson-Yeo et al. (2014) lies in the combination frameworks employed. Multi-kernel learning algorithms can be divided into one-step methods or two-step methods in terms of their training methodology (Gonen and Alpaydin, 2011). One-step methods using fixed rules and heuristics generally do not have much computational complexity to determine the kernel combination weight, whereas two-step methods update the combination weights by solving an optimization problem whose convergence may be slow or even hard to get for a large number of kernels. Thus, in the kernel combination framework, rather than using either an un-weighted simple sum of kernels approach or a complex two-step learning algorithm (Pettersson-Yeo et al., 2014), we chose a heuristic approach using the accuracy of each feature type in the feature selection procedure as kernel combining weights, a simplification necessary with a large number of kernels. In the classifier combination framework, considering that the SVM classifiers trained using different feature types had different performances, we used a weighted voting approach instead of a simple prediction averaging or majority voting method (Pettersson-Yeo et al., 2014).

A previous work by our group applied a SVM-based classification procedure to rsFC data from 21 smokers and 21 non-smokers. That work mainly focused on testing different characteristics of nicotine dependence related network connectivity to predict smoking status. The classifier achieved an accuracy of 78.6% using within-network functional connectivity measures via LOOCV (Pariyadath et al., 2014). In contrast, the present work focused on methods for combining multiple resting-state feature types using different classification frameworks. Moreover, it was generated from a larger dataset of 100 smokers and 100 non-smokers, which would have been expected to result in a more reliable classification result. Besides the difference in sample size, the difference in classification accuracy may also have been caused by different cross-validation procedures (i.e., LOOCV vs. 10-fold cross-validation). In particular, LOOCV, necessary in that study due to the modest sample size, is known to yield anticonservative (i.e., over-fitting) results (Kohavi, 1995). As such, we believe that the current work represents a better estimate of what is possible with rsFC in a smoking model system.

Subcortical brain areas and cerebellum, prominent in our feature maps, are thought to be involved in functional networks supporting higher-order executive function and top-down control, including in various addiction studies (Hester and Garavan, 2004; Dosenbach et al., 2008; Goldstein and Volkow, 2011). For example, the thalamus, shown as one of the most discriminative regions in many of our feature maps, has previously been shown related to nicotine addiction neurobiology (Rubboli et al., 1994; Stein et al., 1998; Franklin et al., 2007; Hahn et al., 2009; Beaver et al., 2011). Additionally, a key alpha5 nicotinic receptor gene variant is associated with a dorsal anterior cingulate-ventral striatum/extended amygdala circuit that distinguishes smokers from non-smokers and predicts addiction severity in smokers (Hong et al., 2010). Moreover, nicotine improves sustained attention by increasing activation in the thalamus, caudate, and occipital lobe (Lawrence et al., 2002). Additional discriminative regions identified in the present study are located in the prefrontal cortex (PFC), which are known to play a key role in addictive behaviors through regulation of limbic regions and its involvement in higher-order executive functions (Goldstein and Volkow, 2011; Zhang X. et al., 2011).

All of the proposed combination frameworks improved classification accuracy over using a single feature type, and are easily applied to cases of resting-state data classification problems. It is worth noting that other imaging data phenotypes (e.g., gray matter density) or even non-imaging measures (e.g., genetics, behavioral, or personality phenotypes) can be added into these frameworks as feature types. In our hands, the feature combination and the classifier combination framework performed slightly better than the kernel combination when using the AAL template; and the classifier combination framework performed better than the other two frameworks when using ICA generated ROIs, although critically, none of these differences would survive a statistical test. Nevertheless, the present results may usefully guide future studies and encourage the use of whichever method investigators are most familiar with or the simplest approach (i.e., feature combination). However, since we only applied these frameworks to a nicotine addiction case as an exemplar, it is not known if a given framework would clearly outperform the other two frameworks when applied to other classification cases, using other templates for feature extraction or indeed other types of classifier input data (e.g., anatomical measures). Nonetheless, our study is an important contribution to the literature as it informs others facing similar choices.

One limitation of this study is that, like many other methodological studies (for example, Demirci et al., 2008; Yang et al., 2010; Castro et al., 2011; Dai et al., 2012; Jie et al., 2015; Kim et al., 2016), our feature combination methods were only validated using one disease exemplar. Notably, our experimental results on nicotine addiction fell into a moderate accuracy range (subject to neither ceiling nor floor effects), which is consistent with the extant literature using machine learning techniques to predict neuropsychiatric disorders (Orrù et al., 2012; Wolfers et al., 2015; Arbabshirani et al., 2016). Given these results, we believe that the proposed method would have good generalizability to other disease exemplars. Another important limitation of this study is that of a limited data size, as larger data sets are known to improve classifier accuracy. Future studies should address these issues.

## Conclusion

In this study, we proposed three optimized frameworks: feature combination, kernel combination, and classifier combination, which we examined separately, to combine multiple types of resting-state features calculated from categories of local measures and network measures into classification. These frameworks were successfully applied to a nicotine dependence case, demonstrating their efficacy in improving classification performance over using a single feature type. Our proposed frameworks have good generalizability and can be applied to other neuropsychiatric diseases with extended feature types from other data phenotypes.

## Ethics statement

This study was carried out in accordance with the recommendations of NIH Human Research Protection Program (HRPP) policies and human subjects protections regulations with written informed consent from all subjects. All subjects gave written informed consent in accordance with the Declaration of Helsinki. The protocol was approved by the Addictions Institutional Review Board of the National Institute on Drug Addiction and National Institute on Alcohol Abuse and Alcoholism.

## Author contributions

XD and TR designed the study. XD conducted the analyses and drafted the manuscript. All authors gave contribution to the data collection, result interpretation, manuscript revision, and approved the final manuscript.

### Conflict of interest statement

The authors declare that the research was conducted in the absence of any commercial or financial relationships that could be construed as a potential conflict of interest.
