# Supplementary material for: Combining Multiple Resting-State fMRI Features during Classification: Optimized Frameworks and Their Application to Nicotine Addiction
Source: Front Hum Neurosci. 2017 Jul 12;11:362. doi: 10.3389/fnhum.2017.00362 (PMC5506584; doi:10.3389/fnhum.2017.00362)
Supplement: Supplementary file 1 [file Presentation1.pdf]

Supplementary Materials

To test the efficiency of using the AAL template to extract features, we additionally tried network-based ROIs which were generated from group ICA maps described as follows.

Sixteen resting-state networks (RSNs) were selected from a 20-component group ICA decomposition of 36-subject resting fMRI dataset in a previous study (Smith, et al., 2009). The discarded four RSNs were identified as artifactual. Of the 16 spatial maps, four were not categorized in the original study, and here we refer to these as three higher order networks (HONs) and one Visual-4 network (Pariyadath, et al., 2014). These 16 spatial maps were resampled to $3 \times 3 \times 3$ mm^3 voxel size, and thresholded at Z=6 with a minimum cluster size of 50 voxels, resulting in a total of 42 ROIs that served as a network-based ROI template (Fig. S1). The template was applied to feature maps of the local and network measures to calculate the mean values in each ROI. It was also used to extract the mean time series in each ROI to calculate the global measures. The performance of three frameworks using the network-based template is listed in Table S1. It shows that all frameworks performed worse comparing to the use of the AAL template. Though the accuracy of the classifier combination was still acceptable, the performance of the feature combination and the kernel combination decreased dramatically.

References

Pariyadath, V., Stein, E.A., Ross, T.J. (2014) Machine learning classification of resting state functional connectivity predicts smoking status. Front Hum Neurosci, 8:425.

Smith, S.M., Fox, P.T., Miller, K.L., Glahn, D.C., Fox, P.M., Mackay, C.E., Filippini, N., Watkins, K.E., Toro, R., Laird, A.R., Beckmann, C.F. (2009) Correspondence of the brain's functional architecture during activation and rest. Proc Natl Acad Sci U S A, 106:13040-5.

Table S1 Classification results while applying the network-based ROI template to all feature types

	Sensitivity	Specificity	Accuracy	Precision	F score
Feature Combination	52.0%	49.0%	50.5%	50.5%	51.2%
Kernel combination	67.0%	63.0%	65.0%	64.4%	65.7%
Classifier combination	72.0%	68.0%	70.0%	69.2%	70.6%

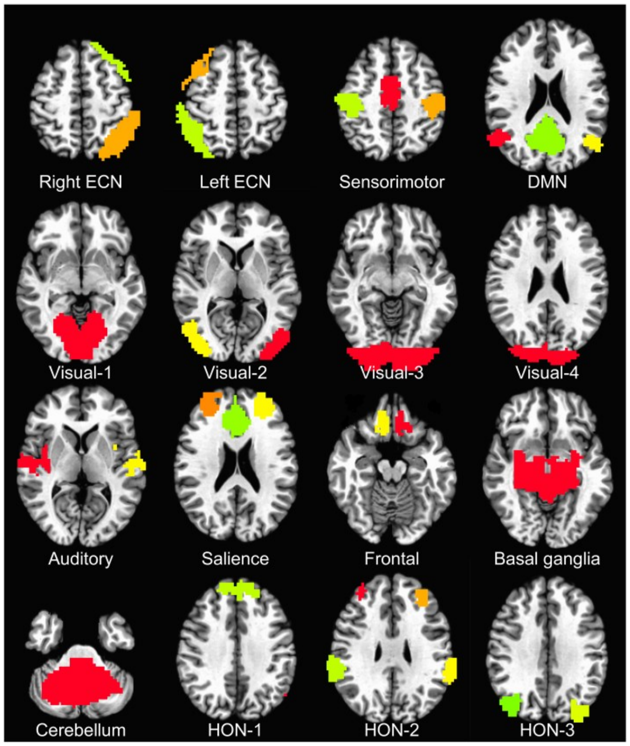

Fig. S1 Network-based 42-ROI template
